# Current Knowledge on Endocrine Disrupting Chemicals (EDCs) from Animal Biology to Humans, from Pregnancy to Adulthood: Highlights from a National Italian Meeting

**DOI:** 10.3390/ijms19061647

**Published:** 2018-06-02

**Authors:** Maria Elisabeth Street, Sabrina Angelini, Sergio Bernasconi, Ernesto Burgio, Alessandra Cassio, Cecilia Catellani, Francesca Cirillo, Annalisa Deodati, Enrica Fabbrizi, Vassilios Fanos, Giancarlo Gargano, Enzo Grossi, Lorenzo Iughetti, Pietro Lazzeroni, Alberto Mantovani, Lucia Migliore, Paola Palanza, Giancarlo Panzica, Anna Maria Papini, Stefano Parmigiani, Barbara Predieri, Chiara Sartori, Gabriele Tridenti, Sergio Amarri

**Affiliations:** 1 Department of Obstetrics, Gynaecology and Paediatrics, Azienda USL-IRCCS, Viale Risorgimento 80, 42123 Reggio Emilia, Italy; cecilia.catellani@ausl.re.it (C.C.); francesca.cirillo@ausl.re.it (F.C.); giancarlo.gargano@ausl.re.it (G.G.); pietro.lazzeroni@ausl.re.it (P.L.); chiara.sartori@ausl.re.it (C.S.); gabriele.tridenti@ausl.re.it (G.T.); sergio.amarri@ausl.re.it (S.A.); 2 Department of Pharmacy and Biotechnology, University of Bologna, Via Irnerio 48, 40126 Bologna, Italy; s.angelini@unibo.it; 3 Former Department of Medicine, University of Parma, Via A. Catalani 10, 43123 Parma, Italy; sbernasconi@gmail.com; 4 ECERI European Cancer and Environment Research Institute, Square de Meeus, 38-40, 1000 Bruxelles, Belgium; erburg@libero.it; 5 Pediatric Endocrinology Programme, Pediatrics Unit, Department of Woman, Child Health and Urologic Diseases, AOU S. Orsola-Malpighi, Via Massarenti, 11, 40138 Bologna, Italy; alessandra.cassio@unibo.it; 6 Department of Pediatrics (DPUO), Bambino Gesù Children’s Hospital, Tor Vergata University, Piazza S. Onofrio 4, 00165 Rome, Italy; annalisa.deodati@opbg.net; 7 Department of Pediatrics and Neonatology, Augusto Murri Hospital, Via Augusto Murri, 17, 63900 Fermo, Itlay; enrica.fabbrizi@libero.it; 8 Neonatal Intensive Care Unit, Neonatal Pathology and Neonatal Section, AOU and University of Cagliari, via Ospedale, 54, 09124 Cagliari, Italy; vafanos@tiscali.it; 9 Villa Santa Maria Institute, Neuropsychiatric Rehabilitation Center, Via IV Novembre 15, 22038 Tavernerio (Como), Italy; enzo.grossi@bracco.com; 10 Department of Medical and Surgical Sciences of the Mother, Children and Adults, Pediatrics Unit, University of Modena and Reggio Emilia, via del Pozzo, 71, 41124 Modena, Italy; lorenzo.iughetti@unimore.it (L.I.); barbara.predieri@unimore.it (B.P.); 11 Department of Veterinary Public Health and Food Safety, Food and Veterinary Toxicology Unit ISS–National Institute of Health, Viale Regina Elena 299, 00161 Rome, Italy; alberto.mantovani@iss.it; 12 Department of Traslational Research and New Technologies in Medicine and Surgery, University of Pisa, Via Roma, 55, 56123 Pisa, Italy; lucia.migliore@med.unipi.it; 13 Unit of Neuroscience, Department of Medicine and Surgery, University of Parma, Via Gramsci, 14, 43126 Parma, Italy; paola.palanza@unipr.it; 14 Laboratory of Neuroendocrinology, Department of Neuroscience Rita Levi Montalcini, University of Turin, Via Cherasco 15, 10126 Turin, Italy; giancarlo.panzica@unito.it; 15 Neuroscience Institute Cavalieri-Ottolenghi (NICO), Regione Gonzole, 10, 10043 Orbassano (Turin), Italy; 16 Department of Chemistry ‘Ugo Schiff’, University of Florence, Via della Lastruccia, 3-13, 50019 Sesto Fiorentino, Florence, Italy; annamaria.papini@unifi.it; 17 Unit of Evolutionary and Functional Biology—Department of Chemistry, Life Sciences and Environmental Sustainability (SCVSA)-University of Parma–11/a, 43124 Parma, Italy; stefano.parmigiani@unipr.it

**Keywords:** Endocrine Disrupting Chemicals (EDCs), neurodevelopment, autism, obesity, puberty, fertility, thyroid function, epigenetics, carcinogenesis, growth

## Abstract

Wildlife has often presented and suggested the effects of endocrine disrupting chemicals (EDCs). Animal studies have given us an important opportunity to understand the mechanisms of action of many chemicals on the endocrine system and on neurodevelopment and behaviour, and to evaluate the effects of doses, time and duration of exposure. Although results are sometimes conflicting because of confounding factors, epidemiological studies in humans suggest effects of EDCs on prenatal growth, thyroid function, glucose metabolism and obesity, puberty, fertility, and on carcinogenesis mainly through epigenetic mechanisms. This manuscript reviews the reports of a multidisciplinary national meeting on this topic.

## 1. Man-Made Environmental Endocrine Disrupting Contaminants: Impact on Wildlife and Human Health

Numerous xenobiotic chemicals used in everyday life and released into the environment by human activity, have the potential to disrupt the endocrine system of wildlife and humans at ecologically relevant concentrations. Of approximately 85,000 known chemical products, approximately 1000 are recognised as potential endocrine disruptors. These include plasticisers as phthalates and bisphenol A, flame retardants, industrial chemicals including alkylphenols, metals and dioxins, air pollutants such as polycyclic aromatic hydrocarbons, and pesticides.

The endocrine system plays a central role in all vertebrates and regulates important biological functions as metabolism, development, reproduction, and behaviour. Since the presentation of the endocrine-disrupting contaminants hypothesis [1] a new emerging science has arisen with concerns relative to the effects of endocrine disrupting contaminants on health and environment [2]. This hybrid multidisciplinary science incorporates findings and methodologies from different disciplines including toxicology, endocrinology, developmental biology, molecular biology, ecology, behavioural biology and epidemiology [2]. An endocrine disruptor is defined as “an exogenous chemical, or mixture of chemicals, that can interfere with any aspect of hormone action” [3]. These chemicals can bind to the body’s endocrine receptors to activate, block, or alter natural hormone synthesis and degradation which occur through a plethora of mechanisms resulting in “false” lack or abnormal hormonal signals that can increase or inhibit normal endocrine function [3]. Data from ecological studies, animal models, clinical observations in humans, and epidemiological studies agree to consider endocrine disrupting chemicals (EDCs) as a significant for wildlife and human health [2,4].

### 1.1. Lesson from Wildlife

Early experimental work was driven by ecological studies that pointed out an association between a complex mixture of xenobiotic pollutants and endocrine disruption of reproduction and development in fish, reptiles (e.g., alligators, turtles), birds, and mammals living in the Laurentian Great Lakes of North America [5,6]. The observed effects suggested estrogenic, androgenic, anti-androgenic, and antithyroid actions. Abnormalities in organs, physiology and behaviour vary from subtle changes to permanent alterations, including disturbed sex differentiation with feminized or masculinized sex organs, changed sexual behaviour, altered immune function, and egg-shell thinning in birds with severe population declines in a number of raptor species in Europe and North America [5,7,8]. Another example are male alligators exposed in ovo (as embryos) to various pesticides which subsequently exhibited significantly reduced plasma testosterone concentrations, aberrant testicular morphology, and small penis size while females exhibited ovarian abnormalities associated with reduced fertility and high embryonic mortality [5,8].

From an evolutionary perspective, the vertebrate-type sex steroid hormones used as regulator of reproduction and development appeared in invertebrate during the evolution of Deuterostomes (Echinoderms and Chordates). Among Protostomes (i.e., Arthropods: insects and crustaceans) steroid molting hormones such as ecdysone have become important regulators of growth, development and reproduction [9]. Several studies have reported reproductive and developmental adverse effects after chronic exposure to EDCs acting as receptor agonist/antagonists of ecdysone and of juvenile hormones [10,11]. Data accumulated over the past two decades reveal substantial global contamination of EDCs by intentional or accidental release into the environment and incorporation into consumer products. An important issue is whether the abnormalities reported in wildlife provide a warning to human health. In this context it should be noted that the vertebrate neuroendocrine system is a clear example of the evolutionary homology principle, as that its development and organization is substantially conserved and similar across the various classes. Indeed, it is not surprising that for centuries wildlife has acted as a sentinel for human health [2,12]. Currently, there are nearly 1000 chemicals reported to have endocrine effects; in addition, new chemicals enter the marketplace each year and the vast majority of them are developed with poor or inappropriate toxicological testing for the detection of potential endocrine disruption [13].

### 1.2. Animal Models: Evidence, Clinical and Epidemiological Studies

There is strong evidence gained from laboratory studies showing the potential of several environmental chemicals to cause endocrine disruption at environmentally relevant exposure levels. Indeed, it is important to underline that similarly to the natural hormones, EDCs can produce profound effects on development at very low dose levels of exposure being pre and early postnatal exposure the most vulnerable periods of life.

Traditional toxicology used doses considered to date as elevated, within the range of parts per million, and evaluated gene mutations, weight loss and death. Current data have evidenced that even very low doses of EDCs (parts per billion and parts per trillion) can cause effects in animals, and several studies have shown in this respect both gene suppression and gene activation [13,14,15]. Given the identification of low-dose effects, which differ from those observed at high doses, the importance of the timing of exposure and the recognition of unique effects during development make previous assumption “the high dose makes the poison” used in risk assessment, simplistic and invalid for many environmental chemicals [2,13,14]. Studies on intrauterine positioning of foetuses in rodents and other animals have proved clearly how low doses of hormones can affect many phenotypes.

In particular, the rodent uterus is an excellent model to study how very low doses of hormones released from neighbouring fetuses can influence the development of endocrine-sensitive morphological and behavioural endpoints in male and female mice [14,16].

Testosterone production in male mice begins around the 12th day of gestation and transfers passively to neighbouring foetuses, so that if a foetus is positioned between two male neighbours it receives higher concentrations of this hormone than a foetus positioned between one male and one female or between two females. Very small differences in testosterone exposure influence then a variety of endpoints including male and female behavioural phenotypes, many of which become apparent only during or after puberty [14,16]. An important issue related to toxicological studies on neurobehavioural effects caused by low dose exposure to EDCs in utero and early development is the lack of evolutionary perspective (i.e., adaptive function). Most of the neurobehavioural endpoints used to study EDC exposure, evolved through Darwinian socio-sexual selection and are sexually-dimorphic therefore, an ethological approach (referred to as ethotoxicological approach) becomes of upmost importance: animals must be tested at different stages of their development, in the appropriate context allowing the functional expression of sex related behaviour/s [17]. For example, bisphenol A (BPA) causes harm in animals at levels to which humans are exposed on average. BPA has the ability to bind to estrogen receptors and initiate cellular responses similar to those caused by estradiol. Recent experiments have shown that at “low doses”, previously predicted to be safe, BPA causes dramatic adverse effects that include chromosomal damage in developing mouse oocytes, and abnormalities in the entire reproductive system in male mice, including a decrease in testicular sperm production and decreased fertility. In this context, EDCs such as phthalates and BPA might be involved in increased hypospadias, male infertility (reduction in sperm counting in the semen), and neurocognitive development [14,18,19]. Epidemiological studies linked EDCs, including dioxins, phthalates, and BPA with reproductive effects, neurobehavioural and metabolic syndrome, bone disorders, immune disorders, and cancers in humans. Animal studies show associations with many additional health effects, including asthma, learning and behavioural problems, early puberty, Parkinson’s disease, breast and prostate cancer, obesity, and other diseases. Indeed, recent epidemiological studies have found significant associations between gestational levels of both BPA and phthalates and cognitive impairment and aggressive behaviours, in animal models [19,20,21] and attention deficit hyperactivity disorder (ADHD) in children [22,23]. The common thread is that exposure to low doses of BPA in utero and during early postnatal life disrupts the development of normal dimorphic behaviours, thus affecting males and females differently [3,15,19,20,21]. Therefore, the most consistent and robust finding across the recent literature on different mammalian species, including humans, is that whenever both sexes have been examined, sex is a fundamental variable in defining BPA effects on behaviour [20,21]. At present, the increase in non-communicable diseases has been related to the exposure to EDCs; these diseases include cancers, endometriosis, infertility, obesity, diabetes, early puberty, susceptibility to infections, autoimmune diseases, ADHD/learning disabilities, neurodegenerative diseases, asthma, and heart disease [4].

### 1.3. Transgenerational Effects, Epigenetics and Sustainability

Recent evidence indicates that exposure to EDCs during development not only can directly harm the exposed individual, but also the individual’s offspring and future generations, a process that is referred to as transgenerational inheritance [24,25]. The data showing these effects led to a new paradigm for non-communicable disease: the developmental origins of health and disease (DOHaD) [4]. The worldwide increase in neurodevelopmental disabilities, including autism, ADHD, infant/childhood depression, social disorders and dyslexia, have been related to industrial chemicals acting as neurotoxicants in the developing brain [26]. Thousands of animal studies show direct causal relationships between a chemical exposure in utero and disease outcomes and in some instances, the adverse effects can be transmitted to subsequent generations through transgenerational epigenetic inheritance (e.g., [24,25,27]). The emerging body of research suggests that exposure to EDCs could have consequences not only for our own health and for that of our children, but also for the health of the generations to come. Indeed, several chemicals, including some EDCs, have the potential to cause health effects in the offspring of exposed individuals through environmentally induced epigenetic modifications. Thus, if we continue to allow human exposure to chemicals with endocrine activity this could affect the sustainability of the wildlife and of the human population. Considered their “stealth” nature, we are currently unprepared to detect the effects of EDCs. EDCs represent one the main factors that can substantially contribute to compromise the sustainability of our environment [1,2]. Therefore, precaution dictates that we cannot wait for “conclusive” evidence of harm to human populations to take action. A more effective communication among scientists, business leaders, regulators, and politicians is required to facilitate science-based decision making.

## 2. Current Knowledge on Exposure to EDCs and Neurobehavioural Development: Lessons from Animals

The increased rates in neurodegenerative diseases, such as Alzheimer’s and Parkinson’s diseases, have been linked to developmental exposure to environmental pollutants [28]. There is strong evidence that one of the most prevalent EDCs, BPA, in addition to causing several adverse effects described in other sections of this review, is a neuroendocrine disruptor at environmentally relevant (within the levels of human exposure) “low” doses and can interfere with sexual differentiation processes in animal models [20,21]. Emerging research on maternal EDC exposure and child neurodevelopmental outcomes have recently found significant associations between gestational levels of BPA or phthalates with alterations of emotional behaviour, aggressive behaviour, cognitive impairment and ADHD in children [20,22]. After more than two decades of experimental research, animal studies have shown that maternal exposure to BPA during gestation and/or lactation induces long-term alterations in offspring behaviour, including mainly three behavioural categories: (1) anxiety and exploration; (2) learning and memory; and (3) socio-sexual behaviours across mammalian species. In addition, treatment with EDCs can also affect mothers’ behaviour. In the following sections, the most relevant findings on EDCs effects on neurobehavioural development in animal models and in epidemiological studies are reported, focusing mainly on BPA, the most studied EDC in the last decade. BPA in the brain has been shown to act primarily as a weak estrogen receptor agonist and as an antiandrogen, and to cause epigenetic changes altering gene expression in different regions [27,29]. We discuss here several studies reporting behavioural effects of prenatal (gestational) and/or postnatal (lactational) exposure to BPA at environmentally relevant “low” doses (below the reference dose of 50 μg/kg bw/day traditionally considered the tolerable daily intake or TDI) via maternal treatment.

### 2.1. Anxiety and Exploration

Despite differences in species, strain and methodology, there is a consistent set of data demonstrating that perinatal exposure to low doses of BPA increased anxiety-like behaviours in different rodent models and using different test paradigms. In particular, BPA exposure reduced exploration and increased anxiety-related behaviours measured through the elevated plus maze test, open-field and dark-light chamber tests in mice [30,31,32,33,34,35,36,37,38], rats [37,38] and other rodent species [39,40].

These BPA-induced effects on anxiety behaviours have been associated with altered mesolimbic dopaminergic signalling, increased expression of glucorticoid receptors in the hippocampus, or with reduction in estrogen-dependent gene expression in the amygdala [32,35,36]. The brain alterations associated with BPA exposure and increased anxiety, are generally sex-dependent and/or alter normal sex differences observed in the control population [20,21,36,37]. Although there are relatively few studies in humans, findings from epidemiological studies are consistent with data in animal models associating maternal BPA levels to internalizing behaviour in children, including anxiety and depression [41,42,43,44].

### 2.2. Learning and Memory

Prenatal and early postnatal BPA exposure were associated with changes in cognitive responses, socio-sexual interactions, play behaviour and parental care in rodents, non-human and human primates [20]. More specifically, impairment in spatial learning and memory have been reported in male deer mice [39], rats [45] and mice [46] perinatally exposed to BPA. In non-human primates, prenatal exposure to low-dose BPA decreased synaptic spine density in the hippocampus and prefrontal cortex. Although the experimental evidence is limited, these studies suggest that BPA may impair memory formation by interfering with neural plasticity processes. Two epidemiological studies have come to conflicting conclusions, one reporting significant maternal BPA-associated cognitive impairment in children [47], the other with no significant correlation [48].

### 2.3. Socio-Sexual Behaviour

With regard to Socio-Sexual Behaviour, a few studies highlighted a reduction of social interactions in BPA-exposed animals; BPA decreased play behaviours in male juvenile cynomolgus monkeys after BPA exposure during gestation, [49] while in rats, reduced female social play [50] and male sexual approach behaviours [51] were reported. A study in mice reported increased play behaviour and social investigation in BPA-exposed juveniles; in addition, the observed effect was transgenerationally transmitted up to the third generation, without further treatment, suggesting an epigenetic effect of BPA exposure via the germ line [27]. During development, rats and mice exposed to prenatal BPA showed decreased oxytocin and vasopressin gene expression that may contribute to explain the observed behavioural changes [27,37]. Thus, age at testing, developmental stage of exposure, sex and other variables can influence BPA effects on social behaviours. With regard to human studies, some evidences indicate sex-dependent associations between gestational BPA or phthalate exposure with alterations in social and aggressive behaviours in children and adolescents [41,42,52,53].

### 2.4. Maternal Behaviour

Direct exposure to EDCs may affect maternal behaviour of treated females and alter the delicate, reciprocal mother-pup relationships [54]. Maternal exposure during pregnancy to a low, but environmentally relevant BPA dose, through a non-stressful administration procedure (i.e., allowing pregnant female mice to drink corn oil in which BPA was dissolved) produced subtle alterations in maternal behaviour and in the behavioural development of their offspring [55]. Specifically, mice fed 10 μg/kg BPA during late pregnancy showed a reduction of subsequent maternal nursing behaviour and an increase of time spent away from the nest over the first 2 weeks post-partum. Further studies in rats [56,57], mice [31,58], voles [59] and California mice [60] confirmed changes in maternal behaviour following exposure to BPA and other EDCs during gestation and lactation. These findings suggested that pregnancy and lactation represented “vulnerable periods of development” for the mother and that maternal brain, physiology and behaviour were highly sensitive to endocrine disruption. Perinatal BPA exposure may also decrease the female offspring engagement in maternal cares in mice and rats [55,56,58] indicating transgenerational impact of BPA exposure on the neuroendocrine substrates modulating maternal behaviour. It is well recognised that in rodents, variation in maternal care per se can affect the offspring growth rate and the subsequent neuroendocrine and behavioural responses that was shown to be associated with epigenetic exposure [54,61]. This implies that an analysis of maternal behaviour should be included, or at least considered as a possible variable, when assessing the effects of chemicals administered via maternal treatment [54].

### 2.5. EDCs Effects Are Sex-Specific

Previously reported studies proved that BPA at low, environmentally relevant doses can affect behaviour in animal models and epidemiological evidence is also growing. Specific effects of BPA on behaviour can vary because of differences in study design, animal models, behavioural endpoints, etc. Recent evidence has repeatedly shown in several mammalians, including humans, that the effects of BPA on behaviour differ in males and females.

Normal sex-differentiated behaviour, with differences observed in males and females are present after exposure to BPA in utero and in early postnatal life [20,21,62]. The most consistent and robust finding across the recent literature on several mammalian species, including humans, is that sex is a fundamental variable in accounting for BPA effects on behaviour. Numerous studies have also confirmed the ability of BPA and other EDCs to affect rodent developing brain in a sex specific way even at very low doses by disrupting normal steroid programming of the brain through epigenetic alterations that can lead to differential gene expression [63]. Unfortunately, it is not clear how the various sex-specific behavioural differences found in rodent models will translate to humans. However, sex specific effects of BPA exposure seem to be a feature also in human epidemiological studies [20,64]. Prenatal BPA levels are positively associated with increased externalizing behaviours in girls [44], increased internalizing behaviours, anxiety and aggression in boys [41,43]. Since many neuropsychiatric disorders show a sex–specific incidence, it is important to unravel how hormones and other factors shape neurobehavioural dimorphisms. An additional consideration is that whenever considering any developmental factor, sexually dimorphic consequences that need to be accounted for. In view of the fundamental and more consistent results linking BPA exposure to behavioural effects by altering brain sexual differentiation, endocrine disruption studies must examine sexual dimorphic behaviours.

## 3. EDCs and Neurodevelopmental Diseases in Humans: Focus on Autism

Disability originating from neurodevelopmental disorders is extremely common affecting more than 10% of children [65,66]. The most common neurodevelopmental disorders include learning disabilities, sensory deficits, developmental delays, attention deficit and hyperactivity disorder and autism, which is the most severe and costly [67] due to the associated permanent disabilities.

The causes of autism spectrum disorder (ASD) remain elusive despite a large amount of basic and clinical research performed over the last ten years. There are consistent reasons to think that ASD is already present at birth; several neurological changes have been reported to develop during fetal life in response to various and heterogeneous factors. The role of genetic abnormalities in autism has stimulated a huge amount of research, however, the final scenario does not satisfy the expectation. Many twin and family studies point out the importance of inherited predisposition to the disorder although epidemiologic research suggests the strong contribution of prenatal and early postnatal environmental factors. Indeed, genetic factors alone account for approximately 20–30% of all cases, whereas 70–80% are the result of complex interactions between environmental risk factors and inherited or de novo genetic susceptibility [68]. Though the prevalence of autism is undoubtedly increasing over time [65], it is not clear if this increase is due to diagnostic improvement or to a greater susceptibility of the population to this disease. Recent studies point to an equal contribution of environmental factors, particularly environmental toxicants, and genetic susceptibility [69]. Only few industrial chemicals (e.g., lead (Pb), methylmercury, polychlorinated biphenyls (PCBs), arsenic (As), and toluene) are recognized causes of neurodevelopmental disorders and subclinical brain dysfunction. The recent discovery that heavy metals such as cadmium (Cd), As, mercury (Hg), nickel (Ni), and Pb may exhibit endocrine-disrupting activity in animal models, probably by interfering with zinc-fingers of nuclear estrogen receptors [70].

### 3.1. Hg

Hg represents the most studied compound in relation to the risk of autism. A recent review [71] considered studies, published between 1999 and 2016, examining the potential relationship between Hg and ASD, including studies on Hg levels in human tissue, biomarkers for Hg exposure, and epidemiological studies. Referring to this comprehensive review, four studies reported that some brain auto-antibodies correlated with Hg levels in children with ASD, finding that is biologically plausible since previous studies reported that exposure to Hg, in particular to the Hg-based compound Thimerosal, caused autoimmune dysfunction [72,73,74,75].

Several epidemiological studies were carried out to check if thimerosal in vaccines was a risk factor for ASD, the majority confirming that thimerosal in vaccines was a risk factor for ASD, a minority found no associations [71,76,77,78,79]. A number of studies examined susceptibility to Hg (or “pro-oxidant environmental toxins”) in ASD. These studies used a variety of tissues, including brain tissue, lymphoblastoid cell lines (LCLs), and blood samples [71,80,81,82]. A major focus was trans-methylation/trans-sulfuration concentrations, which were consistently found to be abnormal in ASD [83,84]. It was concluded that children with ASD had increased oxidative stress and reduced detoxification capacity due to limited thiol availability and decreased glutathione (GSH) reserve capacity [71,85,86,87].

Furthermore, a few human tissue studies described in blood (whole blood and red blood cells) and nails, higher Hg levels in those with the worse symptoms [88,89,90].

Increased urinary coproporhyrin (cP), pentacoproporphyrin (5cxP), and the presence of precoprpophyrin (prcP), an atypical porphyrin, which is not found in the urine of unexposed controls, proved Hg toxicity and Hg body burden. Finally, relationships between ASD severity and porphyrin biomarkers of Hg exposure have been described [71,91,92].

The presence of Hg in air pollution has also been regarded as a risk factor for ASD [71,93,94].

In conclusion, the vast majority of these studies suggested Hg as an ASD risk factor, describing both direct and indirect effects. The preponderance of the evidence indicates that Hg exposure is causal and/or contributory to ASD.

### 3.2. PCBs

PCBs have the strongest and longest-known associations with neurological disorders. In humans, there is evidence for impaired neurodevelopment, lower intelligence level (IQ), and problems with attention, memory, and fine motor skills such as writing. PCBs have been recognized as persistent organic pollutants, and for this reason were banned in almost all countries many years ago. However, due to their lipophilic nature, PCBs have bio-accumulated in the food chain, and currently PCBs levels are still measurable in blood samples, including those from pregnant women, and breast milk samples. In a recent population-based case-control study in southern California 11 PCB congeners were measured in banked second trimester serum samples relative to ASD (N = 54), intellectual deficit (ID) (N = 181), and general population (GP) controls (N = 418) [95]. ASD risk was elevated for a number of PCB congeners, particularly for those showing a concentration within the highest quartile (AOR = 1.79, 95% CI 1.10, 2.71). For all these compounds, the first evidence of a potential neurotoxicity came from the detection of acute adverse effects on the adult nervous system, at high doses, followed by case reports and epidemiological evidence on developmental toxicity at lower doses, to which children were exposed. Exposure to these chemicals during early fetal development can cause brain injury at doses much lower than those affecting adult brain functions.

### 3.3. Polycyclic Aromatic Hydrocarbons (PAHs)

Recently, a considerable amount of research has studied whether PAHs, the main air pollutants, are harmful for the brain. Undoubtedly, the most important study on PAHs is the american study on Childhood Autism Risks from Genetics and the Environment (CHARGE study). This study assessed residential traffic exposure in a group of children with autism diagnosed between 24 and 60 months of age (N = 304) and in normally developing matched controls (N = 259). Children allocated in the highest quartile of exposure for the average concentrations of several pollutants, including nitrogen oxides, PM10, PM2.5, and nitrogen dioxide (NO_2_), during the entire duration of pregnancy and the first year of life had a higher risk of autism compared to those in the lowest quartile [96]. Additionally, the distance from a freeway, a significant source of air pollution, was more likely to be smaller in cases compare to controls (≤309 m) [97].

### 3.4. Polybrominated Diphenyl Ethers (PBDEs)

Since the 1970s to reduce the risk of combustion, synthetic flame retardants as PBDE have been used extensively and have accumulated ubiquitously in the environment. PBDE has been detected in human serum, placenta, adipose and liver tissue, cord serum and breastmilk besides in-house dust, soil, sewage sludge and wildlife [98]. The impact of prenatal and postnatal PBDE exposures on child behaviour has been investigated by a number of epidemiologic studies, recently reviewed, reporting conflicting results [99]. Some of the studies showed a positive association between serum and milk median levels of PBDEs and abnormalities in children behaviour, while others failed to show any associations. Despite these conflicting results, it was concluded that prenatal and postnatal PBDE exposure affected adversely externalizing behaviour (e.g., hyperactivity and conduct problems). Therefore, additional studies are needed to determine whether PBDEs are associated with internalizing problems, adaptive skills, and social behaviours/ASD in children.

### 3.5. Phthalates

Phthalates, ubiquitous contaminants, are used as plasticizers, solvents and additives in many consumer products (i.e., vinyl flooring, wall coverings, food containers and cosmetics). In particular, di-(2-ethylhexyl) phthalate (DEHP) represents one of the most commonly used plasticizers in pharmaceutical and medical devices. A recent systematic review on the association between prenatal and/or childhood exposure to phthalate and ASD highlighted the existence of a limited number of studies on the topic, as only seven were considered of relevance [100]. Of these, two did not measure phthalate exposure, therefore did not yield quantitative results, whereas the remaining five studies measured phthalate exposure in biological samples. Two were cohort studies, one reporting a positive association and one with unclear results; three were case-control studies, two reported a significant correlation between exposure to phthalate and ASD, while the third bared negative results though it showed a compromised phthalate metabolite glucuronidation pathway, as a possible mechanism for ASD.

### 3.6. BPA

BPA is another ubiquitous xenobiotic agent suspected to cause adverse effects on human health. This common plasticizer is used in the manufacturing of polycarbonate plastics and polyvinyl chloride (PVC), as an antioxidant in some plasticizers, and in epoxy resins used to coat the inside of many food and beverage cans. Only one study has specifically addressed the possible association between BPA and autism [101]. In this study, urine specimens were collected from 46 children with ASD and 52 controls. Total BPA concentration, determined by mass spectrometry, was 3 times greater in the ASD group compared with controls suggesting an association between BPA and ASD.

### 3.7. Pesticides

Pesticides are neurotoxic, and associations with ASD symptoms, organochlorine (OC), organophosphate (OP), and pyrethroid pesticide exposure during pregnancy have been reported. The most important study in this context is the CHARGE study [102]. The aim of this study was to investigate if residential proximity to agricultural pesticides during pregnancy was associated with ASD or developmental delay (DD).

The California Pesticide Use Report (1997–2008), linked commercial pesticide application data to the addresses of 970 participants during pregnancy and aggregated pounds of active ingredient applied for OP, OC, pyrethroids, and carbamates with 1.25-km, 1.5-km, and 1.75-km buffer distances from the homes. The study concluded that the risk of ASD increased by 60% in those exposed to organophosphates during gestation and that the risk was greater if this occurred during the third-trimester (OR = 2.0; 95% CI: 1.1, 3.6) or the exposure was to chlorpyrifos during the second-trimester (OR = 3.3; 95% CI: 1.5, 7.4).

Children of mothers living just before conception or during the third trimester near areas where pyrethroid insecticide was used, presented a greater risk of both ASD and DD (ORs ranging from 1.7 to 2.3). The risk for DD was increased in particular in those living close to zone where carbamate was used, but no specific vulnerable period was identified. These evidences strengthen the evidence linking neurodevelopmental disorders with gestational pesticide exposure, particularly to organophosphates, and provide novel results relative to associations between ASD and DD and exposure to pyrethroids and carbamates.

## 4. EDCs and Metabolism

In the last decade emerging evidence has indicated a role for EDCs in the etiology of obesity [103]. In May 2014 a workshop held in Parma produced The Parma Consensus Statement proposing the Metabolism Disruptor Chemicals (MDCs) hypothesis, which postulates that many endocrine disruptors have the ability to promote obesity, diabetes, fatty liver and/or alterations in lipid and glucose metabolism in humans and animals [4]. Overall, these metabolic alterations may play an important role in the global epidemics of obesity, type 2 diabetes (T2D) and Metabolic Syndrome (MetS). It is important to point out that food intake and exercise play an essential role in controlling body weight, but many EDCs can act as MDCs and alter the set-point for gaining weight. Experimental data have shown that EDCs exposure during development can act at different levels on multiple tissues and pathways to increase food intake and metabolism, leading to weight gain by altering the set-point of sensitivity to develop obesity and associated metabolic disorders [4,26]. Indeed, BPA, for example, not only can cause weight gain but also lead to glucose intolerance, T2D and fatty liver in mice [104]. The same has been noticed for some phthalates and tributyltin [26]. By definition EDCs interfere with hormonal actions and sex hormones influence body adiposity and show changes in the metabolic syndrome [105]. Sex specific effects are expected for many EDCs [4], and in fact sex biased effects of developmental exposure to BPA or other EDCs on body weight and metabolic functions have been reported depending upon type and dose of the tested chemical, the timing of exposure and the metabolic challenge [26].

## 5. MDCs and Neuroendocrine Circuits Controlling Food Intake and Energy Metabolism

MDCs are compounds characterized by several peripheral targets (e.g., fat tissue, liver, pancreas, skeletal muscle, intestine), that may also act at the level of hypothalamic neuroendocrine circuits [26]. The hypothalamus (with some structures in the brainstem, as the nucleus of the solitary tract), plays an important role in energy balance regulation and food intake, with two distinct populations of neurons located in the arcuate nucleus (ARC), exerting opposite effects on food intake and energy metabolism [106,107]. One group of neurons expresses the orexigenic neuropeptides Y (NPY) and Agouti Related (AgRP), and receptors for peripheral hormones signalling the energy status of the body (insulin, leptin and ghrelin). An increase in NPY/AgRP release results in increased food intake and decreased energy expenditure. Other ARC neurons produce the neuropeptide melanocyte-stimulating hormone (MSH), which derives from pro-opio-melanocortin (POMC), and the neuropeptide Cocaine- and amphetamine-regulated transcript (CART), both involved in peripheral energy status signalling. In particular, the release of MSH/CART decreases food intake and increases energy expenditure.

Peripheral signals (hormones like insulin, leptin and ghrelin, in addition to sensory nerve fibres) carry information concerning energy stores, food processing, and gastrointestinal activity. The current hypothesis is that as fat tissue increases, both insulin and leptin levels increase along with MSH expression, while NPY synthesis and release are inhibited, resulting in a decrease in food intake. On the contrary, when NPY synthesis and release are increased and MSH is decreased, there is an increase in food intake. Dysfunction of the NPY system has been implicated in obesity and T2D in humans [108]. Both the neuronal systems located in the ARC nucleus send their fibres to the hypothalamic nuclei, are important for metabolic control, the most important being represented by one the paraventricular nucleus (PVN). The two most important hypophysiotrophic systems regulating body metabolism are located in the PVN: the Corticotropin releasing hormone (CRH) neurons, controlling the hypothalamus-hypophysis-adrenal axis (HPA) [109] and the Thyrotropin-releasing hormone (TRH) neurons, controlling the hypothalamus–hypophysis–thyroid axis (HPT) [110]. The MSH system, is sexually dimorphic with females having increased responsiveness to leptin and decreased responsiveness to insulin in comparison to males [111]. The NPY/AgRP circuit is also sexually dimorphic. In particular, NPY immunoreactivity is sexually dimorphic in the ARC, the dorsomedial hypothalamus, and the PVN [112] and the Neuropeptide Y receptor Y1 (NPY-Y1) expression is higher in females compared to males [113]. Both peripheral (e.g., estrogens) and central hormones cooperate in the control of these two main circuits, resulting in the balance between anabolism and catabolism, and the stimulation or the repression of food intake [114]. All the components of these systems (neuropeptides, receptors, signalling molecules) may be targets of the MDCs action, however, only a few studies have investigated alterations of neural circuits/cells in relation to feeding behaviour and energy balance output [26,115]. Among the most studied MDCs are BPA and Tributyltin (TBT). The exposure to low doses of BPA in mice during the prenatal period alters food intake during puberty and in adulthood, as well as leptin and insulin levels, which in turn regulate the NPY system [104]. In addition, prenatal treatment with BPA has a sexually differentiated organizational effect on the MSH and NPY systems [116]. Interestingly, these differences are evident only if adults are exposed to a high-fat diet. Under these conditions, male mice showed reduced MSH fibre innervation of the PVN and increased NPY/AgRP mRNA in the ARC, while females showed reduced POMC mRNA in the ARC, reaching a level similar to that observed in males, suggesting a masculinizing effect of BPA.

With regard to TBT, it is one of the organotin compounds well known for its obesogenic effects, mediated by the Peroxisome Proliferator Activated Receptor Gamma (PPARγ) receptors on the fat tissue [117]. Different studies on mice showed that prenatal exposure to TBT induces hypothyroidism in the progeny; in pregnant females a dose-dependent increase in Triiodothyronine(T3)-independent TRH transcription levels was observed [118,119]. Another study showed that acute exposure to TBT resulted in an activation of neurons in a crucial region for the regulation of food intake, the ARC, thus suggesting a direct action of this compound on the nervous system [120]. Chronic exposure to TBT induced, in adult male mice only, profound alterations of the leptin-NPY-NPY-Y1 system [89], and of the POMC system [121]. Moreover, in adult rats, exposure to TBT induced a sexually expression of mRNA for NPY and POMC [122]. An additional study in rats demonstrated that TBT induces a functional dissociation between CRH, Adrenocorticotropic hormone (ACTH) and corticosterone, and an increase in the expression of nitric oxide synthase in the hypothalamus [123]. The specific action of TBT on the NPY and MSH systems is probably linked to the expression of PPARγ receptors in both neuronal types [124]. In conclusion, despite the limited number of studies on the effects of MDCs in the regulation of food intake and metabolism, the neuroendocrine circuits implicated in their control are important endpoints for the obesogenic action of these compounds, probably mediated by the interaction with PPARγ receptors.

## 6. Effects of EDCs on Glucose Metabolism and Obesity

Obesity, Insulin Resistance (IR) and T2D are related metabolic disorders with a prevalence that has dramatically increased worldwide and at any age over the last decades. The etiology of these conditions is multi-factorial, with lifestyle and genetic background playing a dominant role. However, in recent years, experimental and epidemiological data from the literature suggest an important contribution of EDCs in the onset of obesity and on glucose metabolism impairment as in part detailed in the previous paragraph. Purpose of this paragraph is to present the clinical evidence in this field, summarizing the mechanisms involved and the main epidemiological studies.

### 6.1. The Obesogenic Hypothesis

The term “obesogenic” related to EDCs was first developed in 2006 by Grün and Blumberg ([103]; see Section 4). Studies have shown how EDCs may alter energy homeostasis both in cellular and animal models and in humans, although experimental data seem to be more consistent compared to epidemiological data. EDCs actions involve several mechanisms: increase in number and size of adipose cells, impairment of endocrine regulation of adipose tissue and adipocytokine production, reduction of basal metabolic rate, changes in the regulation of appetite and satiety. These effects are due to molecular actions of EDCs on cellular function via interaction with steroid receptors and nuclear transcription factors, impairment of endocrine signalling transduction and epigenetic mechanisms. In vivo and in vitro models mainly studied the following EDCs actions: interaction with PPARγ and Retinoid X Receptor (RXR), anti-androgenic/xeno estrogenic action and interaction with HPT axis. PPARγ is a nuclear transcription factor, which plays a crucial role in adipocyte biology and is considered the principal regulator of adipogenesis [125,126]. PPARγ acts as a heterodimer, associating with RXR, and regulating the expression of genes involved in adipogenesis and adipocyte differentiation from stem cells [127]. In vivo and in vitro models have confirmed the capacity of EDCs to induce adipogenesis and lipid storage in adipose tissue via interactions with PPARγ [103,128,129,130]. EDCs interaction with PPAR -RXR may finally contribute to the development of the pro-inflammatory status and imbalance in adipocytokine production typical of obesity and of the metabolic syndrome [131,132]. EDCs exhibit anti-androgenic and xeno-estrogenic actions (well described in other sections of this review); androgens and estrogens are involved in the regulation of lipid and glucose metabolism and in the regulation of adipose tissue also [133,134]. Therefore, EDCs may exert their obesogenic action inhibiting the androgen receptor pathway, enhancing the estrogen pathway or reducing androgen conversion through the up-regulation of the aromatase enzyme [135,136,137]. Thyroid hormones have a pivotal role in the regulation of basal metabolic rate and energy expenditure [138]. The role of EDCs in the development of metabolic disease may therefore be related, at least in part, with the disruption of the HPT axis [139,140,141], discussed in a following section (see Section 8). In addition to these well-known mechanisms, recently, other actions of EDCs have been highlighted having a possible implication in the development of obesity. EDCs have been shown to influence the function of metabolic physiological defences against oxidative stress [142], thus enhancing the low-grade inflammatory *milieu* of obese subjects. Finally, in recent years, several studies have suggested a contribution of gut microbiota which can be influenced by EDCs [143]. Both the gastrointestinal tract and its microbiota are likely to be exposed to EDCs through the diet. EDCs dietary exposure have been shown to alter the composition of microbiota. These changes are associated with abnormalities in the host gut immune homeostasis with subsequent changes in cytokine production and hepatic lipid and glucose metabolism [144]. Table 1 summarizes the principal obesogenic EDCs and their mechanisms of action.

### 6.2. Diabetogenic Hypothesis

Over the last decade, there has been a huge increase in the prevalence of T2D. This epidemiological trend is consistent with the exponential increment in the production of synthetic chemicals, an evidence that induced some authors to consider the possibility of a role of EDCs as diabetogenic compounds, regardless of their influence on adipose tissue metabolism [145]. Diabetogenic compounds may exert their action both impairing insulin production at the pancreatic beta cell level and disrupting insulin sensitivity in peripheral tissues. EDC actions on pancreatic function can occur through different mechanisms; for examples, TBT reduces beta cell mass and enhances beta cell apoptosis [146]; phthalates reduce beta cell insulin content [147]; BPA impairs insulin secretion [148]. With regard to peripheral tissues, EDCs reduce insulin sensitivity acting on insulin targets, particularly in the liver. In animal models, BPA alters hepatic glucose sensing, impairing glucokinase (GCK) specific activity [149].

### 6.3. Trans-Generational Effects of EDCs and Metabolic Disturbances

Effects of EDCs on adipogenesis and glucose metabolism may not limit to directly exposed individuals. A huge amount of data emerged during recent years regarding trans-generational actions of EDCs through the epigenetic modulation of regulatory networks. Indeed, animal models have shown that BPA, TBT, pesticides and phthalate exposure determines an increase in the prevalence of obesity and reproductive disease up to the third generation [150,151]. Skinner and his group clearly demonstrated that this effect is secondary to epimutations in a network of genes known to be associated with obesity and its complications [152]. Epigenetic modifications, including abnormal DNA methylation have been also identified in genes involved in insulin sensitivity, such as the GCK gene, after ancestral exposure to EDCs [153].

### 6.4. Evidence in Humans

Although experimental animal models confirm a profound impact of chemical pollutants on adipocyte physiology and glucose metabolism, evidence in humans is still scarce, with data often conflicting. The reasons of the discrepancies are complex, probably the results of different factors, including intrinsic features of each EDC, variability of EDCs distribution in the environment, differential actions of EDCs, depending on the developmental time-window of exposure and concomitant exposure to a mixture of chemicals, with a likely synergistic effect, known as cocktail effect phenomenon [154]. This complexity renders difficult to build a strong epidemiological model to study the mechanisms of action of EDC in humans and to understand the real clinical impact of each EDC. Moreover, the main publications in the field regard cross sectional or case-control studies. Longitudinal studies are still very limited; therefore, it is necessary to confirm or strengthen data derived from experimental models and cross-sectional studies. Table 2 reports the main evidences concerning the impact of EDCs on obesity and glucose metabolism impairment in humans.

## 7. Effects of EDCs on Prenatal and Postnatal Growth

Fetal growth restriction and premature birth have been associated with EDC exposure.

Exposure during critical periods of development, such as fetal and early postnatal life, may have consequences. This is of importance for research, patient care, prevention and public health [164]. As EDCs are widely distributed in the environment, the majority of pregnant women in the United States have detectable levels of multiple EDCs in their blood or urine [165,166]. Some epidemiological studies have reported the correlation between prenatal exposure to EDCs and infant birth outcomes but the results of these epidemiological studies are contradictory. Many investigators have explored the relationship between EDC exposure and birth weight [167]. Lenters et al. examined 17 chemicals (six phthalates, eight Perfluoroalkyl substances (PFAS), two PCBs and one oral contraceptive pill (OCP)) using Elastic Net Regression analyses highlighting previously unknown relationships between 4 of these EDCs and birth weight: two phthalate metabolites (MEHHP, MOiNP), perfluorooctanoic acid (PFOA), and p,p’-dichlorodiphenyl dichloroethylene (p,p’-DDE) were most consistently predictive of term birth weight. In an adjusted, unpenalized regression model of the four exposures, a 2 SD increase in natural log–transformed MEHHP, PFOA, and p,p’-DDE was associated with lower birth weight: −87 g (95% CI: −137, −340 per 1.70 ng/mL), −43 g (95% CI: −108, 23 per 1.18 ng/mL), and −135 g (95% CI: −192, −78 per 1.82 ng/g lipid), respectively; and MOiNP was associated with higher birth weight (46 g; 95% CI: −5, 97 per 2.22 ng/mL) [167]. A meta-analysis conducted on European birth cohorts, examining occupational EDC exposures using a job exposure matrix, found that pregnant women exposed to more than one EDC class were more likely to have a low birth weight infant [168]. There is also sufficient evidence that increased PFAS, especially Perfluorooctanoic acid (PFOA), exposure is associated with low birth weight, whereas mixed results are reported for other EDCs and birth weight [167,169]. For example, a meta-analysis, described an association between PCB 153 and low birth weight [169] whereas Lenters et al. [167] did not find any association. Similarly, inconsistent associations exist for phthalates and BPA [170,171,172], OCPs [167] and PBDEs [173,174]. In the following paragraphs, we provide an overview on the exposure to the main EDCs, such as BPA, Persistent organic pollutants (POPs) and PBDEs during intrauterine growth.

### 7.1. PBDEs

Flame retardants as PBDE are used in many consumer products such as polyurethane foams used in furniture, mattresses, carpet pads, automobile seats, styrene plastics used for electrical appliances and flame-retardant textiles [175]. The insulin-like growth factor (IGF) system is required for fetal growth and a few studies have suggested that PBDE has the capacity to disrupt this system [176]. A 1 mg/kg prenatal exposure per day of BDE-99 has been described to induce an increase in IGF-I gene expression in the uterus in rats [177]. In humans, only two studies have addressed these relationships, one reporting in 149 women from Taiwan a positive association between BDE-196 in breast milk and IGF-I levels in cord serum and negative relationships of IGF-I with BDE-99 and other 86 compounds [178]. The second study reported a positive correlation between umbilical cord serum PBDE levels and placental IGF binding protein 3 (IGFBP3) gene expression among Chinese children living in one of the world’s largest electronic waste sites [179]. Nine epidemiological studies assessed the relationship between PBDEs and birth weight; six of these reported a negative association (four significant and two non-significant), two others reported no statistically significant association, and one study suggested a negative association in male infants and a positive association in females [174,180,181,182]. A further study described an association between increased PBDEs in breast milk and adverse birth outcomes, including low birth weight, short birth length and chest circumference [183]. Furthermore, a prospective Chinese birth cohort reported that maternal BDE-28 and BDE-100 were negatively associated with birth length, and birth weight, in males only [184]. On the contrary, the Canadian birth cohort GEStation Thyroid and Environment (GESTE) study did not find an association between PBDE exposure and birth outcomes [173].

### 7.2. BPA

Many studies investigated the effects of BPA exposure on the fetus during pregnancy. Despite the efforts, the relationship between BPA and fetal or neonatal growth indexes are inconsistent, and there is not enough evidence to clarify if exposure to BPA during pregnancy affects only fetal growth at the time of exposure, or if it affects postnatal growth also. BPA can readily cross the placenta, and some in vivo experiments have demonstrated that it can cause adverse birth outcomes in offspring. For example, oral administration of 10 mg/kg/day of BPA to pregnant rats caused a decreased number of neonates and survival rate. In utero or neonatal exposure to BPA can alter offspring phenotype by stably altering the epigenome, an effect that can be counteracted by maternal dietary supplements [185]. A study measuring BPA levels in maternal blood and umbilical cord blood showed an increased risk for LBW, and an adverse action of leptin and adiponectin in male neonates in the highest quartile of maternal BPA exposure [186]. Furthermore, in 80 matching samples of pregnant women higher unconjugated BPA exposure levels during first trimester and term were associated with sex specific reduction in birth weight and increase in gestational length [187]. In the Mothers and Children’s Environmental Health (MOCEH) study, a total of 788 mother-child pairs in the third trimester and 366 pairs in the neonatal period who completed BPA assessment and fetal/children growth outcomes were included [188]. BPA measurements were conducted twice in the third trimester, using maternal urine, and once in neonatal urine. The study suggested that BPA exposure was negatively associated with intrauterine linear growth. In particular, 1 log-transformed unit of BPA/Creatinine increase of maternal urinary BPA concentration in the third trimester was associated with a decreased femur length. In addition, 1 log-transformed unit of BPA/Creatinine increase of prenatal BPA concentration resulted in increased weight at birth.

### 7.3. POPs

PFASs and OCs are persistent, bio-cumulative chemicals that have been detected in maternal blood during pregnancy and in cord blood at delivery. PFASs and OCs may act as EDCs, and in utero exposure to these xenobiotics may have consequential developmental effects on the fetus. Animal studies indicate that maternal PFAS exposure is associated with reduced fetal growth. However, the results of human studies are inconsistent. A recent systematic review evaluated the data of 14 selected studies, 8 of which reported an in utero exposure [189]. Measures of birth weight showed a continuous decrease after PFOA exposure although the importance of the association varied and many results were not statistically significant.

Results relative to associations between Perfluorooctane Sulfonate (PFOS) exposure and birth weight were also inconsistent. Higher PFOS and PFOA concentrations were reported overall to be associated with an average decreased birth weight in most studies, however, few data were significantly different. Another study measured perfluorohexane sulfonate (PFHxS), PFOS, PFOA, and perfluorononanoate (PFNA) in 1202 mother-child pairs; overall, PFAS concentrations were not associated with birth outcomes. Only PFOA, PFHxS, and PFNA showed weak, non-significant associations with reduced birth weights ranging from 8.6 g to 10.3 g per doubling of exposure [190]. In this context, considering the discrepancies between the studies, the impact of PFASs on public health is unclear, but undoubtedly, the global exposure to PFASs warrants further investigation [191].

## 8. Effects of EDCs on the Thyroid Gland

Thyroid hormones (THs) are critical for normal growth and neurodevelopment, thus, it is important that thyroid function must be maintained within normal physiological limits both during prenatal and postnatal life. There is growing evidence that EDCs can disrupt thyroid homeostasis, even though the most important knowledge on this topic derives from animal studies, while clinical studies are still few and controversial [192]. The control of thyroid function involves a dynamic interaction among the hypothalamic releasing hormone TRH, the pituitary Thyroid Stimulating Hormone (TSH), and the TH that exist in two major forms: Thyroxine (T4) and T3. The thyroid gland synthesizes THs principally in the form of T4, a pro-hormone. In target tissues, deiodinases 1 and 2 convert T4 into biologically active T3. The principal role of T3 is to regulate target gene transcription via its nuclear receptor.

### 8.1. Iodine Deficiency

**Figure 1:**
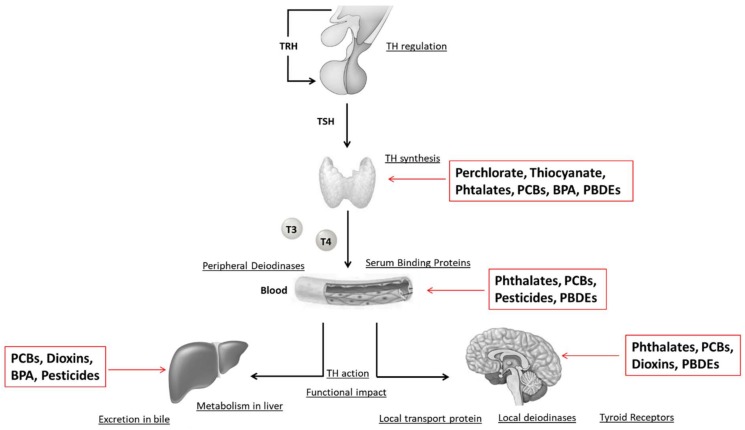
Action of EDCs on the HPT Axis. The black arrows indicate the endocrine axis, the red arrows indicate the organs/tissues targeted by the EDCs.

TH production is normally influenced by the contribution of some environmental micronutrients such as selenium and iodine. In particular, the environmental availability of iodine and its active uptake through the sodium/iodide symporter (NIS) potentially constitute the pathophysiological conditions with which several EDCs can interfere. From this point of view, the most recent epidemiological data (Indian Coalition for Control of Iodine Deficiency Disorders–ICCIDD, 2015) showed that there are still areas of mild/moderate ID in European industrial countries also, although globally iodine deficiency (ID) has improved since 1999. Furthermore, some tissues can regulate their own sensitivity to THs by changes in the expression of various enzymes and transporters [192]. This leads to a situation in which changes in TH action in specific tissues and cells does not reflect changes in circulating levels of THs. Therefore, the evaluation of this parameter is not always reliable to highlight the clinical effects of EDCs, and probably may explain some of the controversial results in the literature observed to date [192,193]. Finally, THs are required throughout fetal life and early childhood for proper brain development. In humans, the fetal thyroid gland does not develop until the second trimester of pregnancy. Therefore, the developing fetus is completely reliant on the maternal source of THs during the first half of pregnancy [194]. In particular, the contribution of THs to the fetus is guaranteed by T4 readily crossing the placenta. In this context, even subtle changes in thyroid function of pregnant women are critical for brain development during fetal life and can cause detrimental effects for the foetus. Therefore, the feto-placental unit may become a target for the action of EDCs [194,195]. There is growing evidence that the HPT axis may be targeted by EDCs, widespread in the environment. Thyroid disruption by EDCs can occur at any level of the HPT axis including TH synthesis (perchlorate [196], thiocyanate [197], phthalates [198], PCB [199], BPA [200], PBDEs [201]), release (phthalates [202], PCB [203], PBDEs [204]), transport (phthalates [205], PCB [203], dioxins [206]), and metabolism (PCB [203], dioxins [206,207], BPA [208]). TH action on target tissues can be disrupted too (PCB [209,210], PBDEs [211]) (Figure 1). Most effects are due, in part, to structural similarities between some EDCs and THs. EDC effects, however, must be evaluated taking into consideration the dependency of the thyroid gland on iodine supply. Likely, the thyroid can adapt partially to adverse EDCs effects, as long as the iodine supply is adequate. As ID still occurs in many countries of the world, this could facilitate and/or enhance the anti-thyroid effects of EDCs. Certain risk groups show a greater tendency to suffer from the consequences of even mild ID, including pregnant and breastfeeding women. Thus, in these groups ID associated with exposure to EDCs may adversely affect the neuro-intellective development of future generations [212].

### 8.2. Perchlorate and Thyocyanate

With regard to clinical studies on EDCs, those concerning perchlorate, thiocyanate and PCB showed evidence of anti-thyroid effects and the possible relationship with iodine supply, prenatal exposure and neuro-intellective development in the offspring [197,213,214,215,216,217,218]. For example, perchlorate and thiocyanate decrease thyroidal iodine uptake by competitively inhibiting the NIS. Exposure to perchlorate and thiocyanate, at least at low levels, occurs ubiquitously as they are naturally found in the environment. In addition, perchlorate is also present in a wide range of products including fertilizers, rockets, fireworks, airbag inflation systems, milk and even prenatal vitamins, whereas thiocyanate is a metabolite of cyanide found in tobacco smoke and increased serum levels are observed in smokers [197]. In this regard, data from National Health and Nutrition Examination Survey (NHANES) 2001–2002 highlighted that in women with low urinary iodine (<100 µg/L) the association between urinary perchlorate and decreased serum T4 was stronger in smokers than in non-smokers and in those with high urinary thiocyanate levels. The authors suggested that the thiocyanate content in tobacco smoke interacted with perchlorate diminishing iodine uptake and affecting thyroid function. In addition, authors claimed that this effect could take place at commonly occurring perchlorate exposure [197]. In a cross-sectional study conducted in 200 first-trimester pregnant Thai women, environmental exposure to perchlorate was found positively associated with TSH and negatively associated with free T4. Thiocyanate is 15 times less potent than perchlorate as iodide competitor for NIS and it is probably for this reason that thiocyanate exposure was positively associated with TSH only in a subgroup of pregnant women with low iodine excretion [213]. Finally, a recent European study conducted as part of a randomized controlled trial on antenatal thyroid screening, reported a relationship between perchlorate exposure in first-trimester pregnant women with low urinary iodine (median 72 µg/L) and measures of reduced cognitive function in the offspring at 3 years of age [214].

### 8.3. PCBs

PCBs and their hydroxylated metabolites are biologically active, accumulate in lipid tissues, and are structurally very close to T4. PCBs may interfere with TH homeostasis in different ways: by binding to transthyretin, by affecting the expression of TH-responsive genes and by antagonizing the complexes from the TH-responsive elements [193]. Many studies in the literature show that both prenatal and perinatal PCB exposure are associated with a variety of cognitive deficits in children. It should be emphasized that the levels of exposure in some of the most recent studies are lower than in the earlier one, yet they reported a negative impact on cognitive function [192,215,216,217]. The literature regarding the relationship between PCB exposure and thyroid function evaluation in humans are controversial. PCBs are complex mixtures of various congeners, each with its own unique molecular structure and potentially different toxic effect. Despite the advances in analytic methods to study these mixtures in human tissue and environmental media, many difficulties remain in identifying reliable markers of the effect of these EDCs on thyroid function [192,215].

## 9. Effects of EDCs on Puberty

Environmental factors have been thought to account for the secular trend in pubertal timing observed in several countries. Menarcheal age has been approximately 13 years for decades, whereas 200 years ago, it was 17 years [218]. Pubertal timing has been recognized as an endpoint possibly influenced by exposure to EDCs and increasing exposure to these compounds has been suggested as a possible factor accounting for the anticipating onset of human puberty [219,220]. The process by which puberty occurs is primarily regulated by the activation of the hypothalamic-pituitary-gonadal (HPG) axis and HPA axis through their chemical messengers, specifically the sexual hormones [221]. These axes are under the control of both inhibitory and stimulatory mechanisms [222]. Disruption of this system by exposure to environmental hormone-mimicking substances may profoundly affect pubertal development. The prevailing opinion on EDCs and puberty is that changes in pubertal timing consist predominantly in an anticipation of female puberty [219,220]. However, both girls and boys appear to experience changes in pubertal timing. A negative distortion of age distribution towards younger ages for initial pubertal stages is observed in both sexes as well as a positive distortion of age distribution towards older ages for the completion of puberty [218,223]. EDCs have many mechanisms of action. Many EDCs are known to act as agonists of estrogen receptors or to antagonize androgen receptor; progesterone receptors are also a potential target for many chlorinated EDCs [224]. Therefore, EDCs may mimic naturally occurring estrogens and androgens in the body or they may potentially cause overstimulation of hormonal pathways. In addition, EDCs might bind to a receptor within a cell and block the functions of endogenous hormones, acting as antiestrogens and antiandrogens [225,226]. In humans, it is difficult to provide evidence of a causal relationship between changes in pubertal timing and EDCs exposure [218]. Specifically, causation is difficult to demonstrate on the account of exposure to low doses of tenths or even hundreds of chemicals starting in prenatal life. An additional and critical concern is the potential lag between exposure, mainly in early life, which is particularly sensitive to EDCs effects, and observation of potential consequences on pubertal timing. Previous Expert Panel and Endocrine Society Scientific statements reviewed the literature on human studies that assessed associations between EDCs exposure and puberty timing [222,227]. The major studied EDCs with regard to puberty include pesticides (dichlorodiphenyl trichloroethane (DDT) and its primary metabolite DDE), polybrominated flame retardants (polybrominated biphenyls (PBB), PBDE), dioxin, phthalate esters, and BPA.

### 9.1. Chlorinated Pesticides–DDT and DDE

With regard to pesticides, the main data came from the observation of early or precocious puberty in children migrating to Belgium, for international adoption, formerly exposed to the estrogenic insecticide DDT in the country of origin (Asia, Africa, and South America) via their biological mothers, during pregnancy, and directly after birth. Median DDE concentrations were significantly higher in adopted (*n* = 15/40) and non-adopted (*n* = 11/40) foreign-girls with precocious puberty with respect to Belgian native girls with idiopathic or organic precocious puberty who showed detectable concentrations in 2 out of 15 cases. Moreover, DDE levels were positively correlated with age at immigration and negatively correlated with time since immigration [228,229]. The authors hypothesized that emigration may interrupt exposure to DDT and precocious puberty could result indirectly from withdrawal of the negative feedback of the sex steroids and their environmental analogues and/or directly from accelerated hypothalamic maturation caused by sex steroids. Despite the associations found in this study, the conclusions remain speculative; data on migrating children showing early or precocious puberty depict the concept that environmental clues affect the timing of puberty differently, depending on the life period when they come into action [218]. In the Michigan angler cohort study, including 213 female offspring, in utero exposure to DDE was estimated using a decay model based on maternal measurements. A significantly earlier menarche was observed among girls with an increased in utero exposure. Specifically, menarche was 1 year earlier for every 15 µg/L increase in in utero exposure to DDE [230]. Possible mechanisms for DDE effects include androgen blocking, estrogen-mimicking effect or induction of aromatase. However, data are discordant, as none of these studies associated puberty-timing measures, Tanner stages, and age at menarche with either in utero or lactational DDE exposure [231]. A significant dose-response relation between serum DDT concentrations and earlier menarche was also observed in 466 newly married, nulliparous female Chinese textile workers [232].

### 9.2. PBDEs

PBDEs exposure during the peri-pubertal period was suspected to interfere with reproductive development. The association between serum PBDEs and age at menarche was evaluated in 271 adolescent girls in the NHANES 2003–2004. Higher serum PBDEs concentrations were associated with slightly earlier age at menarche: from the first to the fourth quartile of total PBDEs concentrations, a higher percentage of adolescents in the higher PBDEs exposure group experienced menarche before 12 years [233]. In Italy, PBDE serum concentrations were determined in two different studies. A study on 31 girls with idiopathic central precocious puberty showed a median PBDE level of 59 ng/g of lipids [234], one order of magnitude higher than in those reported in a similar German study [235], and less pronounced compared to samples from US girls. However, the upper quartile values were comparable to the serum concentrations and was significantly associated with an increased risk of earlier menarche [233]. In the second study, a case-control study including 37 girls with idiopathic central precocious puberty and 56 with premature thelarche PBDE serum concentrations corrected for total lipid content resulted significantly higher in girls with premature thelarche than in controls and higher than in idiopathic central precocious puberty girls [236]. The effect of in utero exposure to PBDEs on sexual maturation was evaluated in Michigan girls whose mothers were accidentally exposed through diet to these compounds. In particular, pubertal development was assessed in 327 females exposed to PBBs in utero and, in many cases, throughout breastfeeding. Girls who were exposed in utero to high PBDEs concentrations and who were breastfed reported menarche 1 full year earlier than unexposed girls (11.6 years vs. 12.2) or girls who were exposed only in utero (11.6 years vs. 12.7 years). Perinatal exposure was associated with earlier pubic hair appearance in breastfed girls, while no association was found with breast development [237]. These associations support the hypothesis that pre- and postnatal exposure to organhalogens might affect pubertal events. Moreover, considering that menarche and breast development are estrogen-dependent events whereas pubertal hair growth is independent from estrogen levels, these findings suggest that PBBs may interact with different pathways. Recently, associations between prenatal and childhood exposure to PBDE with changes in pubertal timing were studied in a longitudinal cohort study including mainly families of Mexican origin in Northern California. Prenatal concentrations of PBDE were associated with later menarche in girls (RR earlier menarche = 0.5) and earlier pubic hair development in boys (RR earlier pubarche = 2.0). No associations were seen between prenatal exposure and time of girls’ breast or pubic hair development or of boys’ genital development and concentrations [238]. Although published data are conflicting, findings suggested that PBDEs exhibit estrogenic and androgenic properties and ubiquitous exposures may impact children’s pubertal development.

### 9.3. Dioxins

Dioxins are a group of well-characterized endocrine disrupters [239] and exposure to dioxins was the only condition associated with delayed breast development as demonstrated in girls with higher prenatal and lactational exposure in a small (*n* = 18) Dutch cohort study [240]. Slow breast development to the adult stage was also demonstrated in Belgian children and was associated with high dioxin exposure, whereas the age at menarche or pubic hair development showed no correlation with exposure [241]. In 1976, as a result of a chemical explosion, residents of Seveso (Italy), experienced the highest levels of 2,3,7,8-Tetrachlorodibenzo-p-dioxin (TCDD) exposure. Pubertal development was retrospectively examined among 282 women who were exposed post-natally or during childhood and no change in age at menarche has proven to be associated with a 10-fold increase in TCDD serum level [242].

### 9.4. Phthalates

The endocrine disrupting mechanism of phthalates is not fully understood; however, several studies indicate a possible anti-androgenic effect as well as estrogen agonistic and antagonistic activities. In general, phthalates have been associated with earlier puberty, although studies are not in agreement. In Puerto Rico, a temporal trend toward premature thelarche in girls has been noted during the early 1980s [243]. Based on the data accumulated by the Premature Thelarche and Early Sexual Development (PTESD) Registry, the estimated annual average incidence rate of premature thelarche in these Puerto Rican girls was 8 cases per 1000 live female births from 1984 to 1993 [244]. Serum samples from 41 Puerto Rican girls with premature thelarche and 35 controls were analyzed to determine the possible presence of pesticides and phthalate esters. Significantly higher phthalate levels were found among the girls with premature thelarche; specifically, 68% of the girls with premature thelarche had measurable levels of phthalates compared with 14% of control samples. These findings were suggestive of a possible association between phthalate exposure and premature thelarche in girls [245]. The possible phthalate anti-androgenic effects were suggested by a cross-sectional study from 725 healthy Danish girls, where the highest quartile of phthalate excretion was found to be associated with delayed pubic hair development [246]. However, in a subsequent longitudinal study, including both boys and girls, pubic hair occurrence did not appear associated with phthalate, even in the most exposed girls, whereas appeared anticipated in the most exposed boys, who also showed higher levels of testosterone and lower levels of adrenal androgens [247]. Despite Frederiksen and co-authors demonstrated no differences in urinary phthalate metabolite levels between girls with precocious puberty and controls [246], more recently both plasma and urinary phthalate levels were found to be significantly higher in girls with central precocious puberty compared to both peripheral precocious puberty and control groups [248,249]. Finally, in the Breast Cancer and the Environment Research Program (BCERP) Puberty Study, age of menarche was demonstrated to be younger with increasing levels of high-molecular weight phthalate, measured several years earlier [250].

### 9.5. BPA

The ubiquitous use of BPA provides great potential for exposure to its well-known estrogen-like action. A recent review showed that only 7 out 19 analysed studies demonstrated a correlation between BPA and puberty [251]. Conversely, most cross-sectional studies performed in girls with precocious puberty demonstrated that serum or urinary BPA levels were significantly higher than in control girls, suggesting a role for BPA in the etiology of idiopathic central precocious puberty [252,253]. Regarding age at menarche, as endpoint in pubertal maturation, the association with urinary BPA was analysed in the NHANES 2003–2010 study including data on 987 adolescent girls aged 12–19. Adolescent girls with moderate BPA levels appeared to be less likely to have early onset of menarche than those with the lowest levels (OR = 0.57; 95% CI = 0.30, 1.08) [254]. A recent study evaluating 655 girls aged 9–18 years in Shanghai also suggested the association between BPA exposure and delayed age at menarche [255]. Girls with moderate to high BPA urinary levels were more likely to have delayed menarche compared to girls with undetectable BPA. Moreover, among girls with detectable BPA levels, girls aged 9–12 years were more likely to have reached pubic hair stage 2 (onset of puberty), while girls aged >15 years were less likely to have reached pubic hair stage 5 (completion of puberty) [255]. In contrast with previously described studies, a multi-ethnic group of 192 healthy 9-year-old girls did not report any significant associations with breast and pubic hair status in relation to BPA exposure [256]. Similar to the other EDCs, the conflicting results among published studies do not allow the establishment of a clear role of BPA in timing pubertal changes.

## 10. Effects of EDCs on Fertility

In the last 40 years several studies showed a decline in semen quality [257,258], evaluated as significant decrease in total sperm count, motility, viability and normal shape, resulting in a reduction in the chances to procreate [259]. The causes of this decline are still under investigation but it has been suggested that the exposure to environmental chemicals such as EDCs, during intrauterine development and in adulthood, could be a potential cause of male reproductive disorders [260]. Indeed, recent studies reported an increase in hypospadias and cryptorchidism in association with maternal exposure to environmental pollutants [192,261]. In addition, female fertility seems to be affected by exposure to EDCs [261] as reported in epidemiological studies in humans, in animal models and in many in vitro studies [262,263]. EDCs interfere with the steroid hormone levels and alter function and structure of reproductive organs [192]. Epigenetic mechanisms also play a pivotal role in male and female infertility. Indeed, EDC-induced reproductive disorders have been associated with DNA epigenetic modification (mostly DNA methylation) and have been proven in animal models across multiple generations [192,264]. As mentioned above EDCs effects on human fertility are still unclear as shown by the discordant results of the various studies. The timing and dose of EDCs can result in different phenotypes, therefore, investigating the critical exposure window appears to be essential to understand their different effects. Moreover, the human population is exposed to a mixture of EDCs (previously define as *cocktail effect*), making it difficult to study the effect of a single EDC on fertility and increasing the variability of results. The majority of studies has investigated the effect of pesticides, industrial chemicals and related substances (phthalates, BPA, PCB), dioxin and dibenzofurans on EDCs-induced infertility [192].

The main studies reporting on the effects of EDCs on fertility in women and men are reported in Table 3 and Table 4 respectively.

## 11. EDCs and Carcinogenesis

The depiction of cancer as a genetic accident, explained by the so-called somatic mutation theory (SMT), which has dominated this field for some decades, is increasingly questioned for both epidemiological and molecular reasons [289]. Therefore, the terminology still in use–initiation/promotion, and the clear separation among genotoxic and non-genotoxic agents-strictly connected to this specific context needs a critical review. EDCs in adults, have quite different effects compared to childhood and fetal life, and act mostly as morphogens, altering cell differentiation and interfering with the epigenetic programming of cells, tissues and organs, opening the way to chronic inflammatory, metabolic and cancerous diseases. The SMT model becomes questionable. The real limitation of the SMT model is to be a linear, reductionist, mechanistic model. Indeed, in this model, carcinogens are all agents hypothetically endowed with sufficient “power” to directly damage DNA or to disrupt some key cellular metabolic processes in a potentially irreversible way [290], while the substances lacking this “power” may act as cocarcinogens, essentially by promoting the action of carcinogens. On the other hand, in the context of the emerging systemic and dynamic genomic models, all carcinogenic factors act initially through epigenetic mechanisms: hypomethylation of the whole DNA sequence, hypermethylation of the regulatory/tumour suppressor genes, activation of the mobile sequences and the microRNA networks acting as real “sensors” of stress/danger [291]. It is important to emphasize that in this context, mutations should not be considered the cause of cancer, but the consequence of progressive genomic instability induced by a prolonged exposure to many different stressors. In a dynamic model like this one, also the (co)cancerogenic role of many EDCs would be better explained. In particular, an early exposure to these ubiquitous substances, mainly during the prenatal period, could induce the abovementioned reactive, potentially defensive epigenetic changes, while a subsequent interaction with the same (or similar) stressors would determine the neoplastic transformation of this poorly programmed tissue. In fact, within such a model, the Knudson’s “Two-Hit” Hypothesis of cancer causation (hitherto recognized only in some rare forms of cancer) [292] would become a sort of universal pathogenic theory, better explaining why currently all non-communicable diseases are increasing all over the world, and we are observing a continuous anticipation in the age of onset of the damage. This is obviously the context of the DOHaD theory which is essentially based on three closely interconnected concepts: fetal programming, developmental plasticity, epigenetic mismatch [293]. In such a context, cancer (above all, child and juvenile cancer) should be considered as a dysontogenic process [294], like all other chronic diseases-inflammatory, metabolic and degenerative-that are increasing in the world.

Renzo Tomatis, the former Director of the International Agency for Research on Cancer (IARC) and founder of the IARC Monographs program [295], had already proposed this hypothesis over 40 years ago [296], specifically in relation to what is generally considered the world’s first drug disaster, prenatal exposure to diethylstilbestrol (DES). In a recent update after a 40-year follow up, the association between prenatal DES exposure and clear cell adenocarcinoma of the vagina and cervix has been confirmed [297]. At that time, the link between the exposure of pregnant animals to chemical carcinogens and the occurrence of tumours in the progeny was well documented. Scientists had already hypothesized that the increased risk of cancer in DES girls would be due to exposure to stilbestrol during pregnancy [298]. Tomatis showed that the exposure to DES of pregnant rats resulted in an increased incidence of tumours at specific sites in untreated animals of the second and third generations [299]. In those years, epigenetics was only a theory and it was not possible to hypothesize an epigenetic molecular mechanism at the origin of these unexpected cases of transgenerational carcinogenesis. Only after decades scientists could demonstrate that DES is a powerful endocrine disruptor that interferes with the expression of several uterine genes involved in tissue patterning, such as Wnt Family Member 7A (Wnt7a) [300], Homeobox A9 (Hoxa9), Homeobox A10 (Hoxa10), and Homeobox A11 (Hoxa11) [301], contributing to changes in tissue architecture and morphology. With regard to this, recently performed in vitro and in vivo experiments showed that Homeobox C6 (HOXC6) is an estrogen-regulated gene in breast cancer cells which expression may be induced by exposure to estrogenic EDCs such as BPA, in competition with estradiol (E2) [302]. Exposure to E2 or BPA altered the epigenetic status of the HOXC6 promoter, including increased histone H3K4-trimethylation and histone acetylation, ultimately resulting in HOXC6 gene activation [302].

The DES tragedy not only showed the carcinogenic mechanisms of an endocrine disruptor, but also elucidated how the carcinogenic process can begin in the foetus as altered epigenetic tissue programming (first hit/tumour initiation). In the following years, other studies showed how an early-life exposure to DES during development of the uterus may enhance the penetrance of a tumor-suppressor gene defect in the adult and revealed that a second exposure could trigger the following phases of the neoplastic process (second hit/tumour promotion) [303]. With the intention to deepen the mechanism of DES-induced carcinogenesis, a recent study evaluated miRNA expression in the Syrian golden hamster model, receiving DES on the day of birth [304]. In particular, this study highlighted how DES-induced neoplasia in the hamster uterus includes a spectrum of miRNA expression alterations, providing key new insights on the epigenetics contribution to EDCs carcinogenesis.

The fact that the in utero exposure to EDCs predisposes both to neoplastic forms and to genito-urinary malformations demonstrates that the implicated molecular mechanisms are, at least in this early period of life, essentially of epigenetic nature, perturbing cell differentiation and tissue development (fetal programming). An important confirmation of the link between the morphogenetic and the carcinogenic potential of EDCs comes from the numerous studies on TCDD (the so-called Seveso dioxin) [305] already recognized by the IARC as a potent carcinogen [306], and PCBs [307]. These studies showed how TCDD and PCBs act in the fetus by altering the development of the mammary gland, an organ physiologically characterized by a very high degree of plasticity, having to assume various conformations and to perform different activities in different periods of life, particularly during and after pregnancy [308].

It is not surprising that tumours most directly and frequently related to EDCs affect tissues and organs belonging to the endocrine system, in particular the mammary gland [309] and the prostate [310].

Like DES, BPA is an estrogen-like EDC that induces persistent epigenetic changes in the fetus, mainly in the developing uterus and breast. The molecular mechanisms by which epigenetic alterations would produce an increased risk of breast neoplasia after in utero exposure to both molecules have been recently illustrated [311]. A model summarizing the main pathways potentially involved in the BPA action in prostate cancer was recently proposed by Di Donato and co-workers, showing androgen and estrogen receptor mediated gene transcription, contributing to either enhancement or inhibition of cell proliferation. This could occur through epigenetic modifications such as those associated with abnormal activity of histone-modifying enzymes (sirtuins, LSD/KDM lysine-demethylases), recruited to chromatin by steroidal receptors [312]. Another study performed in zebrafish proposed that also the BPA effects on female reproductive function could involve epigenetic mechanism [313]. The inhibitory action of BPA on the ovary could be due to its capacity to down-regulate the expression of the luteinizing hormone/choriogonadotropin receptor (lhcgr) both decreasing and increasing histone methylation and interfering with DNA methyl transferases [313].

Returning to dioxin, it was reported that the pro-lymphomatous chromosomal translocation (t14; 18) increased significantly in normal subjects [314]. Even more interestingly, the same translocation, leading to the continued expression of the anti-apoptotic gene B-cell lymphoma 2 (BCL-2), and thus to the formation of immortalized lymphocyte clones, was described in subjects chronically exposed to pesticides [315]. This clearly shows how totally different molecules, capable of acting as EDCs, can induce the formation of specific chromosomal arrangements, i.e., of reactive and potentially defensive changes in affected cells. The specific modalities of action of EDCs allow and to some extent force us, to consider cancer not a genetic incident, due to stochastic DNA mutations, but the product of a disturbed early epigenetic programming of tissues and organs and of further molecular mechanisms potentially adaptive and defensive towards an environment in continuous and dramatic transformation.

## 12. Conclusions

Most of the knowledge on the harmful effects of EDCs comes from animal studies. Observation of wildlife remains crucial for human health and understanding of the environment, including the effect of chemicals, in particular of those having endocrine and metabolic effects. In recent years, a lot of data has arisen relative to the effects of EDC exposure on metabolism, obesity and its complications, neurodevelopment and behaviour, intrauterine growth, thyroid function, puberty, fertility, and carcinogenesis.

Studies to date underline the brain as a vulnerable target of EDCs. Many of the reviewed studies present significant limitations, including lack of replication, limited sample sizes, retrospective design, publication biases, inadequate matching of cases and controls, and the use of non-standardized tools to diagnose conditions as ASD, although the overall evidence on a pathogenetic role for EDCs is compelling. Experimental animal data show numerous neurobiological changes caused by EDCs, including neuronal development, properties of synaptic organization, neurotransmitter synthesis and release, and structural organizational effects on the developing brain. In addition to this, there is growing evidence on associations between EDC exposure and fetal and postnatal growth, however, we should also underline that findings are often conflicting, and methodological limits are present. For example, with regard to EDCs disrupting thyroid homeostasis, widely diffused in the environment, often it is not possible to find a correlation with circulating TH levels both for methodological issues and for pathogenetic reasons. Some peculiar features of the HPT axis promote the interaction between the environmental supply of iodine and the EDCs antithyroid effects, besides on the neuro-intellective development. This interaction could be particularly significant in high-risk situations and in the most vulnerable groups as in pregnant women, prenatal and perinatal periods.

Multiple lines of evidence suggest a role of EDCs exposures in the modulation of human pubertal timing. However, published data on human pre- or neonatally and postnatally exposed are scarce and no firm conclusions can be drawn. In this context, further studies are needed to address the question of which EDCs mainly affect puberty and how we can reduce relevant exposures. With regard to fertility, studies once again show conflicting results. A possible cause could be the “cocktail effect” and the differences in environment, therefore, further studies are absolutely needed to clarify the role of EDCs on male and female reproductive health.

**Figure 2:**
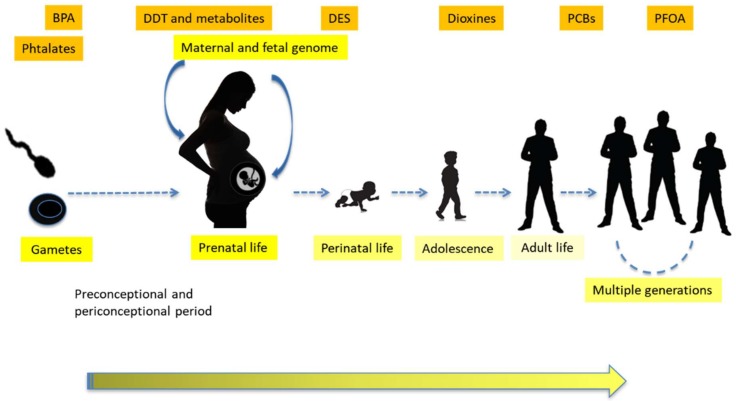
Importance of EDCs driven epigenetic effects during life course and potential consequences across generations according to the Developmental Origins of Health and Disease (DOHaD) theory.

With regard to carcinogenesis, the most powerful pro-carcinogenic mechanisms of endocrine disruptors, seem to be related to their ability to epigenetically interfere with the embryo-fetal programming of tissues and organs (Figure 2). Essentially the proposed embryo-fetal programming occurs by altering the regulation of genes involved in cell cycle, cell proliferation, apoptosis and other key signalling pathways. In view of this consideration, a radical change in the dominant model of carcinogenesis and, to a large extent, in the current pathogenetic models inherent to chronic diseases that are continuously increasing in the world, is likely necessary.

## Figures and Tables

**Table 1:** Principal obesogenic endocrine disrupting chemicals (EDCs) and the site of action.

Chemical	Metabolite	Site of Action
Phthalates	DBP, BP, DHEP	Steroid receptors (anti androgen), PPARs, RXR [128,137]
Phenolic compounds	BPA	Steroid receptors (xeno-estrogen), PPARs, RXR [131,140,141]
Pharmaceutical compounds	DES	Estrogen receptor [136]
Organotin compounds	TBT	PPARs, RXR [129,139]
Dioxins	TCDD	Aryl hydrocarbon receptor [144]
PCBs and POPs	PCB 153-170-187	Aryl hydrocarbon receptor [130,144]
Pesticides	DDT	Steroid receptors [133,134]
Flame retardants	Penta-DBE	Steroid receptors [133,134]
Alkylphenols	NP	Steroid receptors [133,134]

Abbreviations: DBP: dibutyl phthalate; BP: Benzophenone; DHEP: diclofenac hydroxyethylpyrrolidine; PPAR: Peroxisome proliferator-activated receptor; RXR: retinoid X receptor; BPA: Bisphenol A; DES: diethylstilbestrol; TBT: Tributyltin; TCDD: 2,3,7,8-tetrachlorodibenzo-p-dioxin; PCBs: Polychlorinated biphenyls; POPs: persistent organic pollutants; DDT: dichlorodiphenyltri-chloroethane; penta-DBE: pentabrominated diphenyl ether; NP: 4-nonylphenol.

**Table 2:** Impact of EDCs on measures of adiposity and metabolism.

EDC	Population	Endpoint
TCDD (Dioxin)	U.S. Ranch Hand Veterans (Adults)	Increased risk of T2DM [155]
TCDD (Dioxin)	North Italy (Seveso incident, adults)	Increased risk of T2DM (Female) [156]
Persistent Organic Pollutants	Spain (Adults)	Increased risk of metabolic syndrome [157]
Persistent Organic Pollutants	Canada (Adults)	Increased risk of metabolic syndrome [158]
BPA	China (Adults)	Increased BMI, waist circumference and decreased insulin sensitivity [159]
BPA	NHANES (U.S., adults)	Increased BMI and waist circumference [160]
Phthalates	NHANES (U.S. Adults and children)	Increased BMI [161]
Phthalates	NHANES (U.S. Adults and children)	Increased waist circumference, decreased insulin sensitivity (adult males) [162]
Phthalates	Italy (Children)	Increased waist circumference, decreased insulin sensitivity [163]

Abbreviations: TCDD: 2,3,7,8-tetrachlorodibenzo-p-dioxin; T2DM: Diabetes mellitus type 2; BMI: Body Mass Index; BPA: Bisphenol A; BMI: body mass index; NHANES: National Health and Nutrition Examination Survey.

**Table 3:** Recent evidences on the effects of EDCs on fertility in women.

Contaminant	Substrate	Cohort	Results
BPA	Urine	25 Turkish prepubertal girls with premature thelarche (PT), 25 healthy prepubertal girls	The median urinary concentrations of BPA were found to be significantly higher in the PT group compared to the healthy control group, weak positive correlation between uterus volume, estradiol and luteinizing hormone [265]
BPA, phthalates	Urine from mothers during first, second, and third trimesters of pregnancy.	120 female prepubertal subjects	Phthalate metabolites were associated with higher serum testosterone concentrations in prepuberty while a number of metabolites measured in the third trimester were associated with higher DHEA-S. MEHP levels across pregnancy were associated with lower odds of having a Tanner Stage >1 for breast development, while MEHP in the third trimester was associated with higher odds of having a Tanner Stage for pubic hair development >1 [266]
BPA	Urine	268 infertile women diagnosed with PCOS	BPA was detected in all women. Increased BPA correlated with decreased antral follicle count and was negatively associated with AMH and day-3 FSH levels, but neither of these reached statistical significance [267]
BPA	Urine	256 women	No associations between urinary BPA concentrations and IVF outcome [268]
BPA	Urine	143 patients with endometriosis, 287 healthy women	No associations between BPA concentrations and endometriosis. Positive association with non-ovarian pelvic endometriosis [269]
Phthalate metabolites, BPA	Urine	221 women	BPA and MCOP (or its precursors) were associated with shorter luteal phase. DEHP metabolites were associated with reduced early pregnancy loss [270]
Phthalate metabolites	Urine	229 women	No significant association with MBP, MBzP and MEHP. Urinary concentration of MEP was associated with a decreased fecundity [271]
Phthalate metabolites	Urine	128 women	Pregnancy loss was increased in women with urinary increase in MEHP [272]
Pesticides	Follicular fluid	94 women of infertile couples	Higher concentrations of any studied PCBs and pesticides are associated with thinner endometrial thickness and affected embryological ICSI outcomes [273]
Dioxins, PCBs, PBDEs, PBBs, HBCDs, OC pesticides	Adipose tissue and serum samples	55 patients and 44 healthy women	Significant associations between deep infiltrating endometriosis and adipose tissue levels of PCB, PBDE, PBB, benzenes and organochlorine pesticides [274]

Abbreviations: BPA: Bisphenol A; DHEA-S: dehydroepiandrosterone sulphate; MEHP: monoethylhexyl phthalate; PCOS: Polycystic Ovarian Syndrome; AMH: Anti-Müllerian hormone; FSH: Follicle-Stimulating Hormone; IVF: in vitro fertilization; MEP: mono-ethyl phthalate; MBP: monobutyl phthalate; MBzP: monobenzyl phthalate; MCOP: monocarboxyoctyl phthalate; DEHP: di-(2-ethylhexyl) phthalate; ICSI: Intracytoplasmic Sperm Injection; ART: assisted reproductive technologies; OCP: Oral contraceptive pill; PCBs: Polychlorinated biphenyls; PBDEs: Polybrominated diphenyl ethers; PBBs: polybrominated biphenyls; HBCDs: hexabromocyclododecanes; OC: organochlorine; POPs: Persistent organic pollutants; BFRs: Brominated flame retardants; PCDDs: polychlorinated dibenzodioxins.

**Table 4:** Recent evidences on the effects of EDCs on fertility in men.

Contaminant	Substrate	Cohort	Results
BPA	Semen and serum	365 semen samples. Maternal serum collected at 18 and 34 weeks’ gestation	Sperm concentration and motility were significantly correlated with maternal serum BPA levels [275]
BPA	Semen and urine	215 healthy young men (18–23 years)	BPA levels were significantly and negatively correlated with sperm concentration. No significant associations were found among BPA and other semen quality parameters or reproductive hormone levels [276]
BPA	Cord blood	283 neonates	Positive association of BPA levels with testosterone, estradiol, and progesterone levels in boys [277]
BPA, phthalates	Urine from 1st, 2nd, 3rd trimesters of pregnancy	109 boys	Exposure to phthalates during the 3rd trimester associated with lower odds of having Pubic Hair Tanner stage >1 for and higher peripubertal SHBG levels [266]
BPA, PCBs	Plasma and semen	191 men	Seminal BPA, but not plasma BPA, was negatively associated with sperm concentration and morphology. PCB was negatively associated with testosterone, free testosterone, free androgen index and DHT in plasma [278]
BPA	Placenta	28 cases and 51 healthy controls in newborns	Increase of BPA levels are associated with of cryptorchidism and hypospadias [279]
Phthalate	Urine, semen and blood	796 healthy man	Association with low semen quality and alteration of reproductive hormones even with a dose below the reference doses [280]
Phthalate	Serum	112 adolescents	Highest exposure of one DiNP metabolites associated with lower total testicular volume, higher levels of FSH and lower semen volume. Men in the highest exposure of one DEHP metabolite show lower semen volume [281]
Phthalate metabolites	Urine and semen	501 healthy man	Association between urinary metabolites and lower total sperm counts and concentrations, larger sperm head sizes, higher proportions of megalo head sperm morphology. MEHP was significantly associated with higher sperm motility [282]
Pesticides	Blood	189 healthy young men	The total intake of fruit and vegetables was unrelated to semen quality. Intake with low-to-moderate pesticide residues was associated with a higher total sperm count and sperm concentration [283]
Organochlorine Pesticides	Environmental level	963 cryptorchid men; 678 hypospadias; 65 micropenis; 587,142 controls	Prevalence rates for cryptorchidism, hypospadias and micropenis were significantly greater in areas of high environmental exposure to pesticides in relation to those with low exposure [284]
Pesticides (atrazine)	Drinking water	343 cases with hypospadias and 1422 male controls	No association between hypospadias and daily maternal atrazine exposure during the critical window of genitourinary development [285]
Pesticides	Semen	2122 men who underwent andrological investigation for couple infertility	Exposure to pesticides was associated with a significantly higher risk of asthenozoospermia and necrozoospermia [286]
Pesticides	Serum and semen	99 rural and 36 urban men (18–23 years)	Rural men had poorer sperm morphology, higher sperm count, and lower LH levels than urban subjects. Maternal farming during pregnancy was associated with larger anogenital distance and testis volume [287]
PCBs, PCDD/Fs, and PBDEs	Subcutaneous adipose tissue biopsies	44 cryptorchid cases, and 38 controls	Prenatal exposure to PCDD/Fs and PCDD/F-like PCBs may be associated with increased risk for cryptorchidism [288]

Abbreviations: BPA: Bisphenol A; yr: years, SHBG: Sex Hormone Binding Globulin; PCBs: Polychlorinated biphenyls; DHT: Dihydrotestosterone; DEHP: di-(2-ethylhexyl) phthalate; DBP: Dibutyl phthalate, DEP: Diethyl phthalate; DOP: Di-n-octyl phthalate; MBzP: monobenzyl phthalate; MEHP: monoethylhexyl phthalate; DiNP: diisononyl phthalate; FSH: Follicle-Stimulating Hormone; 2,4-D: 2,4-Dichlorophenoxyacetic acid; LH: luteinizing hormone; PCDD/Fs: polychlorinated dibenzo-p-dioxins and furans; PBDEs: Polybrominated diphenyl ethers.

## Abbreviations

ACTH	Adrenocorticotropic hormone (ACTH)
ADHD	Attention deficit hyperactivity disorder
AgRP	Agouti related neuropeptide
AOR	Adjusted odds ratio
ARC	Arcuate nucleus
As	Arsenic
ASD	Autism spectrum disorder
BCERP	Breast Cancer and the Environment Research Program
BPA	Bisphenol A
CART	Cocaine- and amphetamine-regulated transcript
Cd	Cadmium
CHARGE	Childhood Autism Risks from Genetics and the Environment
CRH	Corticotropin releasing hormone
DD	Developmental delay
DDE	Dichlorodiphenyl dichloroethylene
DDT	Dichlorodiphenyl trichloroethane
DEHP	Di-(2-ethylhexyl) phthalate
DES	Diethylstilbestrol
DOHaD	Developmental origins of health and disease
EDCs	Endocrine disrupting chemicals
GCK	Glucokinase
GESTE	GEStation Thyroid and Environment
GP	General population
Hg	Mercury
Hoxa10	Homeobox A10
Hoxa11	Homeobox A11
Hoxa9	Homeobox A9
HOXC6	Homeobox C6
HPA	Hypothalamus-hypophysis-adrenal axis
HPG	Hypothalamic-pituitary-gonadal axis
HPT	Hypothalamus–hypophysis–thyroid axis
IARC	International Agency for Research on Cancer
ICCIDD	Indian Coalition for Control of Iodine Deficiency Disorders
ID	Intellectual deficit
IGF	Insulin-like growth factor
IGFBP3	IGF binding protein 3
IQ	Intelligence level
IR	Insulin-resistance
LCGHR	Luteinizing hormone/choriogonadotropin receptor
MDC	Metabolism Disruptor Chemicals
MetS	Metabolic Syndrome
MOCEH	Mothers and Children’s Environmental Health (MOCEH)
MSH	Melanocyte-stimulating hormone
NHANES	National Health and Nutrition Examination Survey
Ni	Nickel
NIS	Sodium/iodide symporter
NO_2_	Nitrogen dioxide
NOS	Nitric oxide synthase
NPY	Neuropeptide Y
NPY-Y1	Neuropeptide Y receptor Y1
OC	Organochlorine
OP	Organophosphate
OR	Odds ratio
PAHs	Polycyclic aromatic hydrocarbons
Pb	Lead
PBB	Polybrominated biphenyls
PBDEs	Polybrominated diphenyl ethers
PCBs	Polychlorinated biphenyls
PFASs	Perfluoroalkyl substances
PFHxS	Perfluorohexane sulfonate
PFNA	Perfluorononanoate
PFOA	Perfluorooctanoic acid
PFOS	Perfluorooctane Sulfonate
PM10	Particulated matter with an aerodynamic diameter of 10 μm
PM2.5	Particulate matter with an aerodynamic diameter of 2.5 μm
POMC	Pro-opio-melanocortin
POPs	Persistent organic pollutants
PPARγ	Peroxisome Proliferator Activated Receptor Gamma
PTESD	Premature Thelarche and Early Sexual Development
PVC	Polyvinyl chloride
PVN	Paraventricular nucleus
RXR	Retinoid X Receptor
SMT	Somatic mutation theory
T2D	Type 2 diabetes mellitus
T3	Triiodothyronine
T4	Thyroxine
TBT	Tributyltin
TCDD	2,3,7,8-Tetrachlorodibenzo-p-dioxin
THs	Thyroid hormones
TRH	Thyrotropin-releasing hormone
TSH	Thyroid Stimulating Hormone
Wnt7a	Wnt Family Member 7A
